# Monitoring Cropland Phenology on Google Earth Engine Using Gaussian Process Regression

**DOI:** 10.3390/rs14010146

**Published:** 2021-12-29

**Authors:** Matías Salinero-Delgado, José Estévez, Luca Pipia, Santiago Belda, Katja Berger, Vanessa Paredes Gómez, Jochem Verrelst

**Affiliations:** 1 Image Processing Laboratory (IPL), University of Valencia, C/Catedrático José Beltrán 2, Paterna, 46980 Valencia, Spain; 2 Institut Cartogràfic i Geològic de Catalunya (ICGC), Parc de Montjüic, 08038 Barcelona, Spain; 3 Department of Geography, Ludwig-Maximilians-Universität München (LMU), Luisenstr. 37, 80333 Munich, Germany; 4 ITACYL, Agrotechnological Institute of Castile and León, Junta de Castilla y León, Ctra. de Burgos, km. 119, 47071 Valladolid, Spain

**Keywords:** land surface phenology (LSP), Google Earth Engine (GEE), Gaussian process regression (GPR), Sentinel-2, gap-filling, crop traits, hybrid models

## Abstract

Monitoring cropland phenology from optical satellite data remains a challenging task due to the influence of clouds and atmospheric artifacts. Therefore, measures need to be taken to overcome these challenges and gain better knowledge of crop dynamics. The arrival of cloud computing platforms such as Google Earth Engine (GEE) has enabled us to propose a Sentinel-2 (S2) phenology end-to-end processing chain. To achieve this, the following pipeline was implemented: (1) the building of hybrid Gaussian Process Regression (GPR) retrieval models of crop traits optimized with active learning, (2) implementation of these models on GEE (3) generation of spatiotemporally continuous maps and time series of these crop traits with the use of gap-filling through GPR fitting, and finally, (4) calculation of land surface phenology (LSP) metrics such as the start of season (SOS) or end of season (EOS). Overall, from good to high performance was achieved, in particular for the estimation of canopy-level traits such as leaf area index (LAI) and canopy chlorophyll content, with normalized root mean square errors (NRMSE) of 9% and 10%, respectively. By means of the GPR gap-filling time series of S2, entire tiles were reconstructed, and resulting maps were demonstrated over an agricultural area in Castile and Leon, Spain, where crop calendar data were available to assess the validity of LSP metrics derived from crop traits. In addition, phenology derived from the normalized difference vegetation index (NDVI) was used as reference. NDVI not only proved to be a robust indicator for the calculation of LSP metrics, but also served to demonstrate the good phenology quality of the quantitative trait products. Thanks to the GEE framework, the proposed workflow can be realized anywhere in the world and for any time window, thus representing a shift in the satellite data processing paradigm. We anticipate that the produced LSP metrics can provide meaningful insights into crop seasonal patterns in a changing environment that demands adaptive agricultural production.

## 1 Introduction

Monitoring vegetation phenology is vital for understanding the influence of vegetation dynamics on a changing climate [1]. Since climate change is one of the major pressures on agricultural production, assessing the phenology of cultivated lands is becoming increasingly relevant. Phenological data help to trace plant development, monitor agricultural production processes, estimate crop yield [2], and thus ensure food and nutritional security for a growing world population [3–6]. Moreover, the temporal and spatial variabilities of phenology variations help to distinguish different vegetation types [7,8], especially crops [9].

Traditionally, plant phenology is assessed at the ground level and involves visual observations of phenological events, which is labour and time consuming [10]. Therefore, spaceborne observations are employed to monitor the spatiotemporal development of plants at the landscape level, which is also known as ‘land surface phenology’ (LSP) [11]. LSP refers to the seasonal pattern of variation in vegetated land surfaces observed using remote sensing data [12]. LSP metrics are typically associated with general inter-annual vegetation changes that are interpretable from optical remote sensing imagery such as those from the start of greening/season (SOS), the peak of growing season (POS), the onset of senescence or end of the season (EOS), the length of growing season (LOS) [12,13], as well as other transition stages (e.g., maturity, senescence) [14]. These phenological metrics are typically calculated from the normalized difference vegetation index (NDVI) or other common vegetation indices (e.g., [13,15]) and refer to the day of the year (DOY) or the number of days. However, the NDVI approach has some shortcomings, such as a limited sensitivity to the photosynthesis dynamics of vegetation [16]. In contrast, quantitative vegetation variables such as leaf area index (LAI) (e.g., [2,17,18]), when employed to a lesser extent, could support a higher accuracy in the extraction of LSP metrics, in particular for croplands [16,19].

Although the high spatial, spectral, and temporal resolution of the Sentinel-2 (S2) constellation favors cropland monitoring studies [10], modeling the phenological evolution of vegetation traits remains a challenging task. This is mainly due to geometry effects and gaps in the time series caused by clouds and noisy data related to residual calibration and atmospheric correction artifacts [20–23]. Nonetheless, while the delivery of gap-filled, spatiotemporally continuous products is typically beyond the catalogue of products offered by space agencies, the relevance of continuous data streams is well-known.

Since the advent of optical remote sensing science, a plethora of methods for vegetation trait retrieval has been developed. Essentially, quantification relies on a model, linking spectral observations to surfaces variables. While numerous retrieval methods have been presented in the literature (see Verrelst et al. [24,25] for a commonly agreed taxonomy and comprehensive reviews), when it comes to operational and fast processing, preferences tend towards the so-called hybrid methods. Hybrid approaches blend the generic properties of physically based models with the flexibility and computational efficiency of machine learning regression algorithms (MLRAs). Within such a scheme, simulations by a radiative transfer model (RTM) are used to train an MLRA. The MLRA learns the nonlinear behaviour between the pairs of reflectance and vegetation trait of interest, and then predicts the output values for new data based on those learned relationships. Within the diverse families of MLRAs, of most interest are those providing associated uncertainty estimates [26]. In this respect, Gaussian Process Regression (GPR) [27] is particularly attractive as it enables statistical learning developed within a Bayesian framework. Moreover, GPR requires relatively small training datasets while maintaining competitive accuracies [25,28].

In order to make these hybrid methods operationally applicable, of similar importance is the availability of an efficient processing framework. Accordingly, the seamless processing of a vast amount of satellite data in space and time demands: (1) moving towards cloud-computing platforms, and (2) integrating the GPR retrieval algorithms into these platforms. Such a processing scheme can lead to new opportunities towards an interactive on-the-fly processing of crop properties in a cloud computing environment. Recently, the Google Earth Engine (GEE) has emerged as an attractive high-performance computing platform that enables the cloud-based processing of petabytes of satellite data [29]. GEE provides powerful computational capability for planetary-scale data processing and even allows creation and training for well-known machine learning algorithms [30]. Despite the growing capabilities of advanced machine learning tools in the GEE environment, GPR is still absent from their standard libraries. This may be due to the high computational costs and memory demand of these algorithms. Recently, Pipia et al. [31] proposed to introduce lighter GPR models that meet the memory restrictions of cloud platforms. In their study, lightweight hybrid retrieval models were applied to S2 data in GEE, demonstrating the temporal mapping of green LAI over the Iberian Peninsula. This implementation of hybrid models on GEE paved the way toward the mapping of global vegetation properties given the optical data of a selected sensor such as S2 [31]. Moreover, GEE allows us to overcome the most common limitation when dealing with optical data, i.e., cloud coverage, which causes gaps in the data stream. Having easy access to the complete and up-to-date S2 catalogue, the processing of the time series became as straightforward as spatial processing. Hence, by exploiting the temporal domain, robust fitting algorithms can be implemented on GEE that can fill up the gaps. Gap-filling is an umbrella term for time series processing algorithms, which can be broadly categorized into: (1) smoothing and empirical methods, (2) data transformations, and (3) fitting methods [11]. Among these, curve fitting methods are conventionally the most often used, with double logistic curves as a popular method for vegetation seasonality or phenology estimation [32,33].

Analogous to the spatial domain, there is also an ongoing tendency in the temporal domain to use adaptive MLRAs for continuous, gap-filled time series processing. These fitting algorithms (4) possess the ability to learn on their own without being explicitly programmed [34,35]. Among the multiple MLRA approaches currently used for gap-filling, the probabilistic GPR is again appealing, given its flexibility and possibility to deliver associated uncertainty estimates. In this respect, recent studies have demonstrated the efficiency of GPR for LAI time series gap-filling [36–38].

While dealing with a large amount of satellite images in the past was tedious and computationally intensive, processing became more straightforward with the migration of retrieval models and the subsequent processing with GEE. Moving along this line, in principle, cloud-based processing enables the quantification of traits and LSP metrics anywhere in the world given the available satellite data catalogues. The generation of these cloud-free, spatiotemporally continuous data streams is an essential prerequisite for using vegetation products to assess seasonal changes, long-term trends, or abrupt events in crop phenology [33,39,40]. However, an end-to-end workflow that starts from the retrieval of crop products using satellite data, to gap-filling and finally to the computation of LSP metrics within a single processing chain remains to be developed.

Altogether, the above-outlined framework brings us to the main objective of proposing an S2 phenology end-to-end processing chain in GEE that is based on the retrieval of crop traits and gap-filling methods. As a secondary objective, we intended to identify the most suitable crop trait(s) for LSP metric calculation. To achieve this, we implemented the full phenology processing chain on GEE, including: (i) crop trait mapping using hybrid GPR models, (ii) the processing of spatiotemporally continuous maps through GPR fitting, and finally, (iii) the calculation of LSP metrics.

## 2 Methodology

### 2.1 General Concept and Workflow

**Figure 1:**
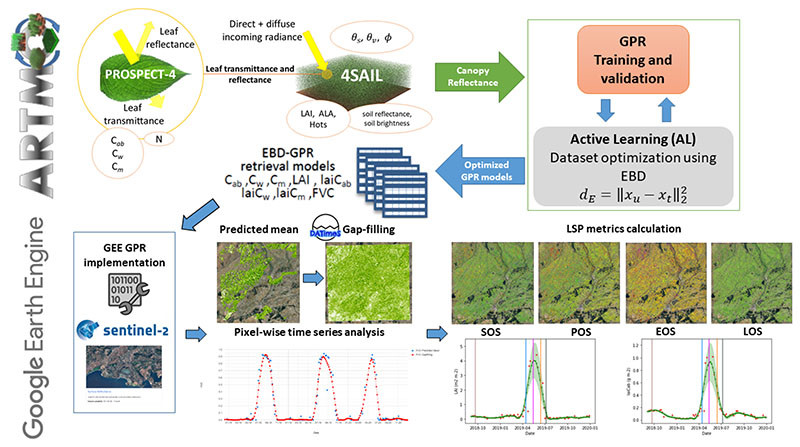
Flowchart of the pursued workflow. Upper: development of hybrid retrieval models in ARTMO by the coupling of leaf-canopy RTMs for training dataset generation, followed by AL optimization and GPR model building. Lower: integration of AL-optimized GPR Euclidean distance-based diversity models (EBD-GPR) on GEE for the retrieval of multiple crop traits from S2 BOA data, subsequent time series gap-filling, and phenology metric calculation.

To build the end-to-end processing chain, we combined different methodologies proposed for the hybrid retrieval of crop traits from S2 Level-2A (L2A) bottom-of-atmosphere (BOA) data [41,42] and the GPR model integration on GEE [31]. The hybrid retrieval workflow with corresponding models is shown schematically in the upper part of Figure 1, while the GEE-based time series processing steps are visualized in the lower part of Figure 1. The main steps are described in the following sections, namely the GPR model theory (Section 2.2), hybrid model development (Sections 2.3 and 2.4), gap-filling and hyperparameter optimization for GEE (Section 2.5), and the processing of time series and LSP metric calculation (Sections 2.6 and 2.7).

### 2.2 Gaussian Process Regression and Adaptations for Processing on GEE

As the core algorithm of this work, the theoretical framework of GPR is first outlined here. GPR is found to be among the preferred kernel machine learning regression models for solving supervised learning problems in GEE. GPR algorithms are straightforward in the training process; they work well with rather small datasets and adopt very flexible kernel functions for establishing nonlinear relationships between spectral observations and variables of interest [43]. Moreover, final retrieval models provide confidence intervals along with the predictions, which give fidelity to the models’ accuracy as well as insights into the robustness of the estimates [26]. An extensive theoretical description is provided in [27], who firstly introduced GPR algorithms, and in the context of Earth Observation (EO) data analysis, descriptions are provided by Camps-Valls et al. [44,45] and Verrelst et al. [24,25]. In the following, we briefly adapt the standard GPR formulation in the spectral domain to crop trait retrieval in GEE and extend the formulation to the time domain for gap-filling purposes.

#### 2.2.1 Standard GPR Formulation

In general, GPR algorithms establish a relation between input data $x\in {\mathbb{R}}^{D}$ and output noisy observations y∈ℝ as *y* = *f* (**x**) + *ϵ*, where *ϵ* is an additive dimension-independent Gaussian distributed noise with zero mean and variance $\sigma_{n}^{2}$, and *f* (**x**) is a Gaussian-distributed random vector with zero-mean and covariance matrix **K**(**x**, **x**), i.e., f(x)∼N(0,K). Each element *ij* of the covariance matrix encodes the similarity between input vectors ***x**_i_* and ***x**_j_* upon disposal, calculated by means of a kernel function *k*(***x**_i_*, ***x**_j_*). Various kernel functions associated with specific degrees-of-freedom, also called hyperparameters, can be employed in a GPR algorithm [27,46]. For vegetation variable retrievals from EO data, the most commonly used kernel is the asymmetric Square Exponential (SE) one, which defines the covariance function as follows:

(1) $$k\left(x_{i},x_{j}\right)=\sigma_{S}^{2}\exp\left(-\frac{1}{2}{\sum_{b=1}^{D}\left[\frac{x_{i}\left(b\right)-x_{j}\left(b\right)}{\sigma_{b}}\right]}^{2}\right),$$

(2) $$\begin{array}{r} f\left(x_{*}\right)=k_{*}^{T}{\left(K+\sigma_{n}^{2}I_{N}\right)}^{-1}y\\ \sigma_{f}^{2}\left(x_{*}\right)=c_{*}-k_{*}^{T}{\left(K+\sigma_{n}^{2}I_{N}\right)}^{-1}k_{*} \end{array}$$

where **k**_∗_ = [*k*(**x**_∗_, **x**_1_), . . . , *k*(**x**_∗_, ***x**_N_*)]*^T^* is an *N* × 1 vector, ***y*** = [*y*_1_, .., *y_N_*]*^T^* and $c_{*}=k\left(x_{*},x_{*}\right)+\sigma_{n}^{2}$.

The probability of the observations given the model’s hyperparameters *p*(***y***|***x***, ***θ***) is given by the marginal likelihood over the function values *f* [27], whose logarithmic expression is as follows:

(3) $$\log p(y|x,f)=-\frac{1}{2}y^{T}{\left(K+\sigma_{n}^{2}I_{N}\right)}^{-1}y-\frac{1}{2}\log|K+\sigma_{n}^{2}I_{N}-\frac{n}{2}\log2\pi$$

The first term in Equation (3) corresponds to a data-fit term, the second term is a complexity penalty, and the last term is a normalizing constant. The maximization of the marginal likelihood, i.e., the minimization of Equation (3), provides the optimum value of **θ**. This optimization procedure is usually referred to as training the GPR [47,48]. Once **θ** has been estimated, the prediction of *y* for a new input vector ***x***_∗_ is given, along with its uncertainty, by Equation (2). Alternatively, the mean predicition can be obtained as a linear combination of *N* kernel functions, each one centered on a training point:

(4) $$f(x_{*})=\sum_{i=1}^{N}\alpha_{i}k(x_{i},x_{*})=k_{*}^{T}\alpha$$

where ${\left\{x_{i}\right\}}_{i=1}^{N}$ are the training vectors contained in the model, *k* is the Kernel function evaluating the similarity between the new input ***x*** and the generic training samples ***x**_i_*, *i* = 1, . . . , *N*, and $\alpha_{i}\in \mathbb{R}$ is the element *i* of the vector $\alpha ={\left(K+\sigma_{n}^{2}I_{N}\right)}^{-1}y$. This last formulation is key to the factorization employed in GEE implementation. Finally, the formulation for the time series domain is straightforward as it is given by imposing *D* = 1: **x** is now scalar and directly indicates the capture time of each time series sample. Substituting **x** for *t*, the hyperparameters of the GPR model retrieving a generic surface property *P_S_* become $\theta_{t}=\left\{\sigma_{st}^{2},\sigma t^{2},\sigma_{nt}^{2}\right\}$, and the corresponding SE kernel for covariance estimation is given by the following equation:

(5) $$k_{t}\left(t_{i},t_{j}\right)=\sigma_{St}^{2}\exp\left(-\frac{1}{2}{\left[\frac{t_{i}-t_{j}}{\sigma_{t}}\right]}^{2}\right),$$

where *t_i_* and *t_j_* denote two generic acquisition dates of non-cloudy acquisitions.

#### 2.2.2 GEE-Integrated GPR Formulation

For the optimal integration of a trained GPR model n GEE, its standard formulation must be reviewed. As seen in Equation (4), the computational burden of GPR predictions grows linearly with the number of training samples, which are represented by **α**. However, each element of this vector can further be factorized into components that depend on: (1) only the training samples, (2) only the prediction input, and (3) a combination of the previous ones. Accordingly, several operations, which are uselessly repeated in the sample-oriented formulation, can be performed only once in a new parallel approach, thus reducing the overall computational burden of the final estimation, as outlined in Pipia et al. [31]. Let $D=diag\left(\sigma_{1}^{-2},..,\sigma_{B}^{-2}\right)$ be the band-dependent hyperparameters of a GPR model trained on a *B*-dimensional input vector, ***X*** = [**x**_1_, **x**_2_, ..., ***x**_N_*] be the *B* × *N* matrix containing its training samples, and *X*_∗_ be the *B* × *M* matrix containing all the pixels of the input *B*-band image to be processed for a new prediction. The matrix $K_{*}=\left[k_{*}^{1},k_{*}^{2},..k_{*}^{M}\right]$, accounting for the similarity between the whole input image ***X***_∗_ and the training information ***X***, can be calculated at once as follows:

(6) $${\text{K}}_{*}=\sigma_{S}^{2}\exp\left(-\frac{{\left(DX_{*}\circ X_{*}\right)}^{T}J_{N,1}J_{1,M}}{2}\right)\circ \exp\left(-\frac{{\left(DX\circ X\right)}^{T}J_{B,1}J_{1,M}}{2}\right)\circ \exp\left(X^{T}DX_{*}\right)$$

where ○ denotes the Hadamard (or element-wise) matrix product, and *J_l_*_,_*_m_* stands for the generic *l* × *m* unit matrix. The prediction for the whole image is finally obtained by parallelizing Equation (4) as follows:


(7) $$f(X_{*})=K_{*}^{T}\left(\alpha J_{1,M}\right)=J_{M,1}\alpha^{T}K_{*}.$$

The same formulation can also be employed for time series gap-filling with minimum modifications. With **t** = [*t*_1_, ...*t_N_t__*]*^T^* being the *N_t_*-dimension date vector of captures available over a specific area of interest, the covariance **K***_t_* of the trained gap-filling GPR model with hyperparameters $\theta_{t}=\left\{\sigma_{st}^{2},{\sigma_{t}}^{2},\sigma_{nt}^{2}\right\}$ is given by the following:

(8) $$K_{t}=\sigma_{st}^{2}\exp\left(-D_{t}\frac{(\text{t}{\text{J}}_{1,N_{t}}-{\text{J}}_{N_{t},1}{\text{t}}^{T})}{2}\right),$$

where $\sigma_{st}^{2}$ is the time series signal variance and $D_{t}=\sigma_{t}^{-2}$. Finally, the mean value prediction at *t*_∗_ over a whole area described by the time series parameter cube ***P****_S_*(***t***) = [***P***_*S*_(*t*_1_), ..., P_S_(*t_N_t__*)]*^T^* is given by:

(9) $$P_{S}(t_{*})={k_{t*}}^{T}{\left(K_{t}+\sigma_{nt}^{2}I_{N_{t}}\right)}^{-1}P_{S}(t)={k_{t_{*}}}^{T}\alpha_{t}.$$

It is worth mentioning that the main hypothesis (i.e., the number of meaningful time samples *N_t_* is equal for all processed pixels) describes an ideal case: whereas the number of acquisitions over the same area is known, the number of non-cloudy samples is pixel-dependent. An efficient workaround to deal with cloudy samples can be found in the work of Pipia et al. [31].

### 2.3 Training Data Generation for Hybrid Model Development

When it comes to time series processing for phenology estimation, we move towards the quantification of crop traits. The pursued strategy to develop hybrid retrieval models can be summarized as follows: the leaf optical properties model PROSPECT-4 [49] was coupled with the canopy reflectance model 4SAIL [50], further referred to as PROSAIL, for the generation of a training dataset. The PROSAIL parameterization information can be consulted in the appendix section (Table A1). By ranging the key PROSAIL input variables according to probability density functions, a random dataset of 1000 simulations of top-of-canopy (TOC) reflectance data was generated according to the 10 and 20 m bands of S2 (10 bands in total). Subsequently, 40 spectral samples from non-vegetated surfaces (e.g., water bodies, bare soil, or man-made) were added to the training dataset. This step is essential to adapt the models to the processing of full heterogeneous scenes, which are typically characterized by various vegetated and non-vegetated areas. Recent studies have suggested that 1000 simulations may be too many for achieving optimal performances, and moreover, the quality rather than the quantity of a training dataset is key to optimizing GPR-based hybrid models [51,52]. Accordingly, the number of samples can be efficiently reduced with active learning (AL) methods. AL provides an optimization strategy by enabling the learner to collect data according to the defined selection criteria [53]. Therefore, the algorithm itself chooses the most representative training samples using an ‘optimal’ statistical approach. As concluded by a systematic literature survey, the Euclidean distance-based diversity (EBD) method is the most accurate and efficient AL strategy for solving regression problems within EO data analysis [31,51,54]. Therefore, we adapted the EBD method for the generation of light GPR retrieval models.

### 2.4 Field Data for Trait Model Tuning and Validation

The retrieval models were trained through AL (EBD) against a validation field dataset collected at the Munich-North-Isar (MNI) test site in Southern Germany (N 48°16', E 11°42'). The MNI site is located within communal farmlands owned by the city of Munich. During the 2017 and 2018 growing seasons, structural and biochemical crop traits were sampled subsequently to field hyperspectral measurements from a winter wheat (*Triticum aestivum*) and a maize (*Zea maize*) field. Extensive descriptions of the campaigns and data collections can be found in Estévez et al. [42], Berger et al. [55], Danner et al. [56], Wocher et al. [57]. Hence, only a brief description is given here. In the test fields, nine elementary sampling units (ESU) of 10 × 10 m were defined and re-visited at two-week intervals. Leaf area index (LAI) measurements, in (m^2^/m^2^), were performed with the LI-COR Biosciences LAI-2200 device. The measurement strategy involved one above-canopy and seven belowcanopy readings, with one repetition at each ESU per date. Note that the optical instrument used is based on gap-fraction, hence the effective LAI was obtained [58]. However, for the sake of simplicity, we will use the term ‘LAI’ throughout the manuscript. In addition, LAI measured by the LAI-2200 device approximates the green LAI simulated by the PROSAIL model.

Leaf chlorophyll content (*C_ab_*), in (μg/cm^2^) was sampled with a Konica-Minolta SPAD-502 hand-held instrument from five leaves per ESU (three points per leaf), while also taking the vertical distribution of the variable into account. Moreover, two leaves were cut at each ESU (18 samples per date). The samples were brought to the laboratory and analyzed using an LI-COR Biosciences LI-3000C scanner for leaf area measurements. Leaf water content (*C_w_*) in (cm) and leaf dry matter content (*C_m_*) in (g/cm^2^) were calculated from the mass difference (per unit leaf size) of sample leaves before and after drying at 105 °C to a constant weight. Lastly, the average value over the nine ESU measurements was calculated for each field sample and considered as representative of a full pixel. Note that the assumption of homogeneity within a pixel may be limited for early growth stages, particularly in the case of maize characterized by pronounced row structures. Nonetheless, the sampling design was adapted to provide representative measurements of all traits over the ESUs, for instance, by taking into account within-row values for LAI measurements [59]. The upscaling of measured leaf biochemicals to the canopy level was performed by multiplication with LAI, resulting in canopy chlorophyll content, i.e., LAI × *C_ab_* (lai*C_ab_*), canopy water content, i.e., LAI × *C_w_* (lai*C_w_*), and canopy dry matter content, i.e., LAI × *C_m_* (lai*C_m_*), all given in (g/m^2^). Note that field measurements of fractional vegetation coverage (FVC) were not available from the campaign.

Subsequently, spectral S2 acquisitions corresponding to the in situ traits were acquired. For that purpose, all available S2 L2A orthorectified BOA reflectance images with a maximum cloud coverage of 1% within the two growing seasons were extracted using the GEE catalog (see Estévez et al. [42]). Note that this low percentage of cloud coverage was only applied to acquire the S2 spectral signatures related to the measured traits at MNI. The developed method can then be applied to regions affected by larger cloud coverage as outlined in Section 2.5. The MNI in situ dataset was used for the optimization process, applying the EBD method to each trait-specific training dataset, i.e., crop trait with corresponding S2 reflectance. Since in situ measurements were missing for FVC, 10% of the simulated data was kept aside for theoretical validation.

To start the AL procedure, an initial dataset of 5% was randomly selected out of the full data pool [52,60]. Then, the EBD methods iteratively selected new samples and evaluated whether they contributed to improving the retrieval model. If the accuracy decreased, the sample was ignored and the algorithm proceeded to the next sample. The processing was repeated until all simulated samples were evaluated against the validation dataset. Finally, trait-specific AL-optimized training datasets were obtained for the building of EBD-GPR models. Thus, each training dataset was composed of a different sample collection and size based on the point of optimal performance. Additionally, the usage of full datasets for model building was tested to evaluate the suitability of the optimized EBD-GPR models for implementation on GEE. For a quantitative comparison and validation, common goodness-of-fit statistics, i.e., the root mean square error (RMSE) in variable-specific units, normalized RMSE (NRMSE in%, which is RMSE divided by a range of observations), and the coefficient of determination (R^2^), were provided.

The development of the hybrid retrieval models was performed with the scientific Automated Radiative Transfer Models Operator (ARTMO, Verrelst et al. [61]) software framework. Within the ARTMO’s MLRA toolbox, an AL module was recently integrated [52]. The described processing steps were therefore based on desktop computers.

### 2.5 Hyperparameter Generation for GPR-Based Gap-Filling

In respect to time series gap-filling, the most important limitations of GPR methods are their (1) memory and (2) high computation time requirements [45] for hyperspectral parameter optimization, growing quadratically and cubically with the number of training points, respectively [62,63]. This can become a serious issue in view of processing a large amount of data, such as in S2 time series tiles. Parallelization strategies for advanced computation platforms such as GEE need to be developed to speed up the GPR processing while maintaining superior performance in terms of accuracy, with these facilities not being devised for per-pixel iterative optimization tasks. To mitigate this computational burden and address repetitive procedures, we pursued the approach by Belda et al. [64]. The study demonstrated that reliable gap-filling could be achieved by making use of pre-calculated hyperparameters (length-scale *l*, signal variance $\sigma_{f}^{2}$, and noise variance $\sigma_{n}^{2}$), which tremendously speed up the training stage of the GPR algorithm (90 times faster than the standard GPR estimations).

First, different crop types were chosen, taking into consideration the available land cover map (i.e., wheat, corn, barley, sunflower, rape, pea, alfalfa, beet, and potato). Secondly, we randomly selected 100 parcels containing more than 50 pixels. Next, for each variable and pixel, hyperparameters were optimally determined by GPR using the conventional per-pixel optimization across the time series. We also estimated a global average of the hyperparameters over all pixels within the randomly selected parcels (i.e., without any crop segregation). To facilitate this process, the three hyperparameters were calculated within the so-called Decomposition and Analysis of Time Series Software (DATimeS) [38], a stand-alone image processing toolbox developed in house and written in MATLAB. Afterwards, the hyperparameters were ingested on GEE for crop trait estimation using Equation (7). The pre-calculated hyperparameters per variable are listed in Table A2. Finally, the GPR models were run over the time series of the crop traits for gap-filling purposes, as formulated in Equation (9). It must be noted that the pursued strategy was not limited to gap-filling, but was also used to apply the fitting function over each pixel in the temporal dimension. Hence, the entire catalogue of generated trait maps were reconstructed according to the GPR fitting model, leading to a spatiotemporal continuous data stream. In addition, the temporal prediction could be produced for any day of the year. Here, it was decided that the crop products would be reconstructed for the same dates as the original acquisitions. Eventually, the spatiotemporal processing was performed at the scale of an S2 tile, making this step the most computationally demanding.

### 2.6 Phenology Metric Calculation with Double Logistics

The following LSP metrics were calculated from the time series: (1) start of season (SOS); (2) end of season (EOS); (3) peak of season (POS), i.e., day when the largest value per season occurs (between SOS and EOS) as well as (4) length of season (LOS), i.e., difference (in days) between SOS and EOS.

The computation of these LSP metrics is typically achieved through a double logistic curve [32,33,65]. This algorithm, also known as Sigmoid, uses a double-sigmoidal model [66] by combining two regular sigmoidal functions:

(10) $$y\left(t\right)=a+\frac{b-a}{\left[1+exp(c+d\cdot t)\right]\times \left[1+exp(e+f\cdot t)\right]}$$

Here, the double-sigmoidal model is uniquely determined by six parameters, two midpoints (*c*, *d*) and two slope parameters (*d*, *e*), a maximum value (*b*), and a base level (*a*). The difference between parameters *b* and *a* gives the seasonal amplitude. To estimate the six parameters on the GEE platform, Li et al. [67] developed a stepwise statistical approach. According to the authors, the performance of this GEE-based double logistic model is robust for different land cover types. A more detailed explanation of this procedure is reported in [68]. Of interest is the fact that this stepwise statistical approach can be implemented at the pixel level on the GEE platform in a parallel manner, which significantly improved the efficiency of large-scale mapping. The phenology metrics of SOS and EOS were derived using a half-maximum criterion method [69]. As such, SOS and EOS were calculated as the dates when the first derivative of the temporal profile reached the maximum increasing and decreasing rates during the green-up and senescence phases, respectively. Although other definitions of SOS and EOS exist, such as inflection points (i.e., at the base of sigmoid curve) [14], the criterion used in our study provides high temporal stability and can be applied to different canopy structures [70].

### 2.7 GEE Implementation and Phenology Metrics Validation

**Figure 2:**
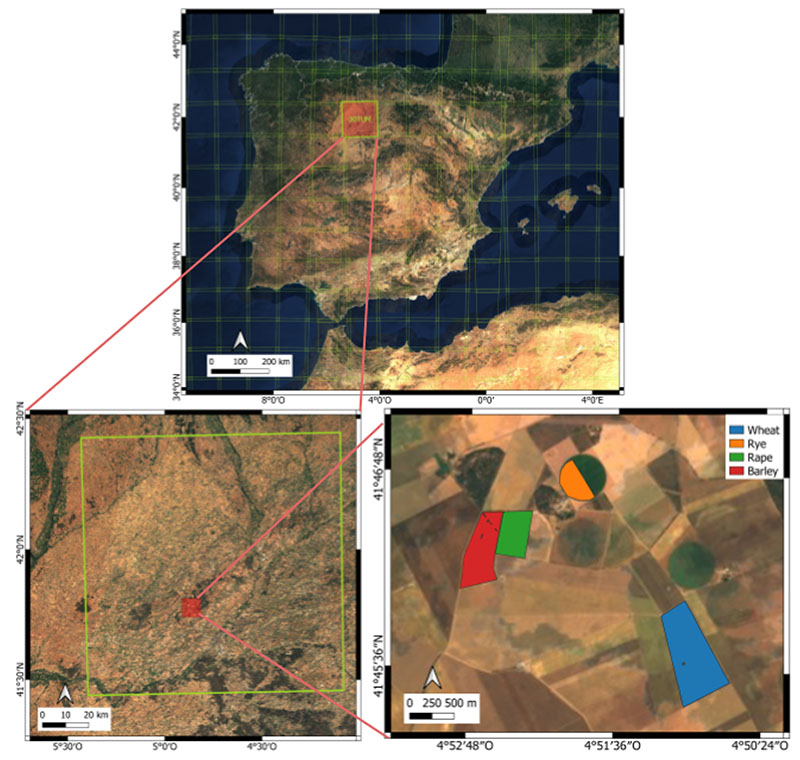
RGB map of the Iberian peninsula (top), the S2 tile 30TUM (Military Grid Reference System) (**left**), and a zoom-in of the crops in the Region Of Interest (ROI) (**right**). Pixels inside the colored region were used for the per-parcel LSP calculations.

Lastly, in order to evaluate the utility of the LSP metrics derived from the crop traits, they were compared against those inferred from the NDVI. Conventionally, the NDVI is used for the calculation of phenological metrics (e.g., [13,15]) and can thus be considered as a reference method. With this inter-comparison exercise, we intend to evaluate the plausibility (suitability) of the different metrics as derived from the two approaches in GEE. Thereby, differences and agreements will be highlighted as a function of crop type. A case study site was selected to demonstrate the developed processing chain of LSP metric derivation. For this, an agricultural region in Castile and Leon in the north-west of Spain was chosen due to the availability of a detailed land cover map. The regional agricultural agency ITACyL (Instituto Tecnológico Agrario de Castilla y León) generates a highly detailed land cover map by using a decision tree-based classifier on satellite imagery time series [71–73]. ITACyL also provided sowing and harvest dates for multiple parcels for the years 2018 and 2019 (over 130 recordings). Given the location of these recordings, the S2 tile 30TUM was processed over the year 2019. Cloud masking was performed using both the SCL and QA60 bands [74]. A zoom-in of the monitored region is provided in Figure 2. Cropland management recordings were available for parcels of wheat, rye, rape, and barley. In summary, the analysis was set up as follows: for each crop trait, the time series of the selected S2 tile was first estimated. Secondly, the gap-filling step was applied, leading to a continuous data stream. Finally, the LSP metrics were calculated. For the selected croplands, mean values and associated standard deviations were derived. These results were then compared to the ground recordings of the sowing and harvest dates, and lastly against the NDVI-based LSP metrics.

## 3 Results

### 3.1 Active Learning Performance for Crop Traits Estimation

**Figure 3:**
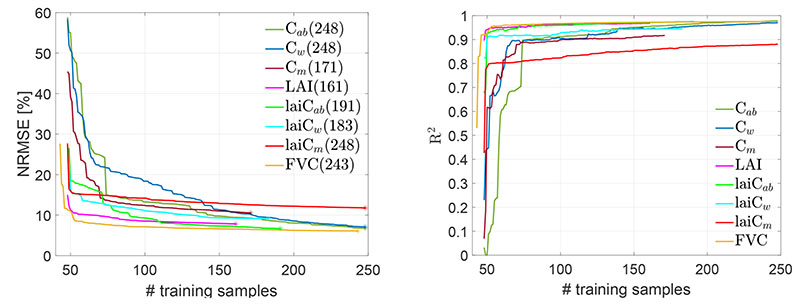
NRMSE (**left**) and R^2^ (**right**) for several trait estimations using the EBD-GPR retrieval model. The optimal number of training samples for the best performance point (marked with an asterisk) is provided in parentheses.

Regarding hybrid methods, training datasets typically contain thousands of samples [42,75,76]. For GPR models, however, this can lead to overly large matrices as they scale cubically with the size of the dataset. To avoid exceeding memory capacity in GEE, light retrieval models should be strived for. As a workaround, the EBD *diversity* method was employed, which helped to identify the most representative training sample for each trait and thus significantly reduced the size of the datasets. Figure 3 demonstrates the results of the EBD method for each crop trait as run against the MNI validation dataset, plus the added 40 non-vegetated spectra to ensure that these spectra were kept in the models. The smoother convergence of the RMSE (Figure 3, left) compared to R^2^ (Figure 3, right) can be explained by the inherent usage of RMSE as a criterion within the AL procedure for keeping or discarding a sample. Nevertheless, the pattern of both metrics is relatively similar for all the variables, triggering a significant improvement in estimation accuracy. The AL procedure started at 48 samples (except for FVC that started at 43 samples), adding a new sample at each iteration, whereby the sample was kept only when it improved the model. This technique led to a rapid decrease of the RMSE and an increase of R^2^, respectively, reaching a stable plateau for the majority of the variables. All in all, only relatively few samples were needed for optimal model performance, ranging from 171 (for *C_m_*) to 248 (e.g., for *C_ab_*), corresponding to 17–25% of the full data pool.

**Figure 4:**
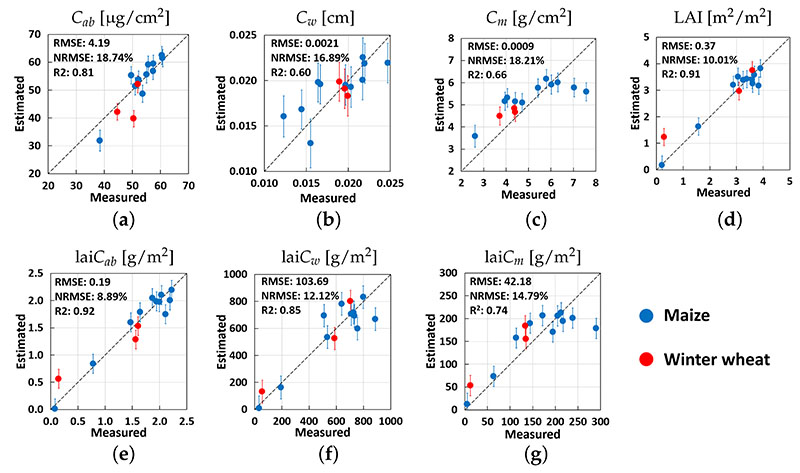
Validation of EBD-GPR model estimates of different crop traits using ground observations from the MNI site (winter wheat and maize) during the 2017 and 2018 growing seasons: *C_ab_* (**a**), *C_w_* (**b**), *C_m_* (**c**), LAI (**d**), lai*C_ab_* (**e**), lai*C_w_* (**f**) and lai*C_m_* (**g**). Measured vs. estimated values are given along the 1:1 line with the associated confidence intervals (1 SD).

Additionally, Figure 4 shows the scatter plots of estimated vs. measured data when selecting the EBD-reduced datasets and validating solely against the MNI validation dataset. Overall, good–excellent retrievals were achieved for all traits. An estimation of canopy-level variables outperformed those of the leaf-level variables, with, e.g., NRMSE = 18.7% and 8.9% of *C_ab_* and lai*C_ab_*, respectively, or NRMSE = 18.2% and 12.1% of *C_m_* and lai*C_m_*, respectively.

**Figure 5:**
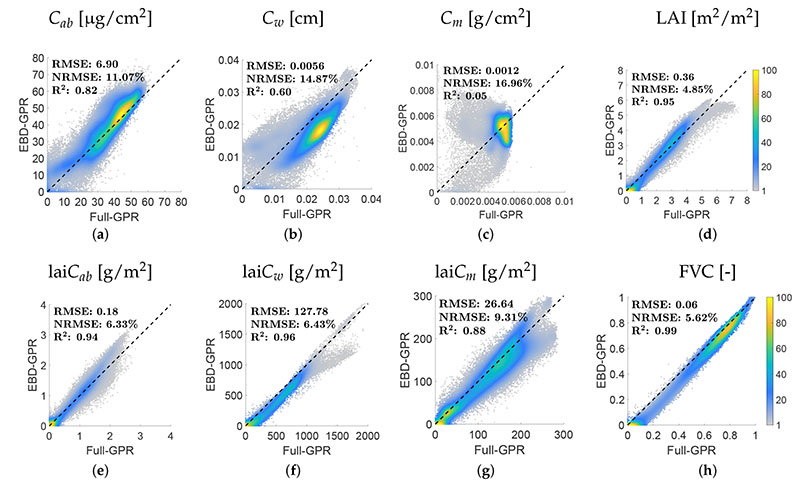
Density scatterplots for comparison of crop trait maps estimated by a full GPR model (not shown) and the EBD-GPR model: *C_ab_* (**a**), *C_w_* (**b**), *C_m_* (**c**), LAI (**d**), lai*C_ab_* (**e**), lai*C_w_* (**f**), lai*C_m_* (**g**), and FVC (**h**). Density in %.

Subsequently, with the aim of assessing whether the reduced models were sufficiently generalizable, they were compared with those obtained from training the full data pools. Table 1 indicates the statistics of observed vs. predicted data of both model types and for each trait. Overall, the EBD-reduced models performed alike or achieved an improvement in estimating the samples of the field measurements. Additionally, in order to assess mapping robustness, both model types were applied and compared for an area of 253 × 315 pixels covering the German MNI location on 6 July 2017. Figure 5 provides the scatter plots between estimated variables using the fully trained and EBD-optimized retrieval models. While the canopy-level variables show consistent correlations (R^2^ > 0.88), the leaf-level traits reveal higher discrepancies. Given the substantial improvements in estimation accuracy (see Table 1), this indicates the efficiency of the AL optimization for this type of traits. Nonetheless, as leaf variables were more poorly estimated than canopy-level traits, they will be excluded from the following analysis. The obtained results for canopylevel variables can be considered as sufficiently precise for a transfer of the EBD-GPR models to GEE.

### 3.2 Crop Mapping and Gap-Filling on GEE

**Figure 6:**
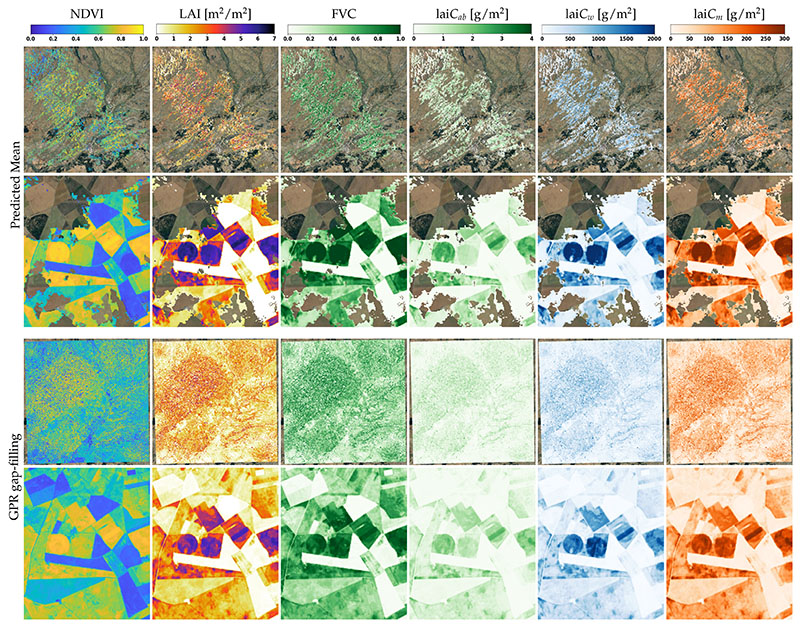
Predicted mean values of NDVI and crop traits for a cloud-gapped tile 30TUM on April 11,2020 (Castile and Leon, Spain) and zoom-in of the study site. Underneath the same maps are shown after GPR gap-filling. See Figure 2 for the geo-information.

Next, the GPR models were integrated on GEE. To demonstrate the functionality, maps were generated for an S2 tile (30TUM) on 11 April 2020 over the case study site in the region of Castile and Leon, Spain (Figure 6, upper). The conversion of the tile into a crop product took about 15 s. Additionally, the NDVI was calculated and mapped on the same tile based on the rationale that vegetation indices–derived LSP is the conventional approach (e.g., [13]). NDVI metrics were therefore considered as reference products in the following analysis.

As the size of an S2 tile is 5491 × 5491 pixels, most of the details are invisible, and the tile is heavily affected by gaps due to cloud cover. Therefore, a zoom-in of the study site was added underneath. For the areas not covered by clouds, the zoom-in reveals that the crop traits were predicted in expected ranges, although with a lack of spatial continuity.

In fact, cloud cover was present on virtually all tiles, causing discontinuous trait maps. This implies that gap-filling became an indispensable step to proceed towards the calculation of LSP metrics. To do so, the time series of S2 tiles were first processed into multiple crop traits with the EBD-GPR models. In total, 73 tiles were processed for the time window between 1 January 2019 and 31 December 2019.

Subsequently, per-pixel temporal processing was applied over the time series of the crop products by means of GPR fitting using pre-calculated hyperparameters per crop trait. Each resulting set of images had a size of 10GB and the processing took approximately 2 h for each crop trait.

Hence, with GPR, not only were the gaps filled, but the full maps for the entire tile were reconstructed using the GPR fitting method. For demonstration, the lower part of Figure 6 displays the spatially continuous reconstructed NDVI and trait maps. The zoom-ins allow us to inspect the reconstruction in detail: gap-filling achieved a meaningful spatial pattern from the agricultural area. Altogether, thanks to the GPR fitting in the temporal domain, spatiotemporal continuous data streams from the tile were produced, enabling LSP metric calculation.

### 3.3 Calculation of LSP metrics

**Figure 7:**
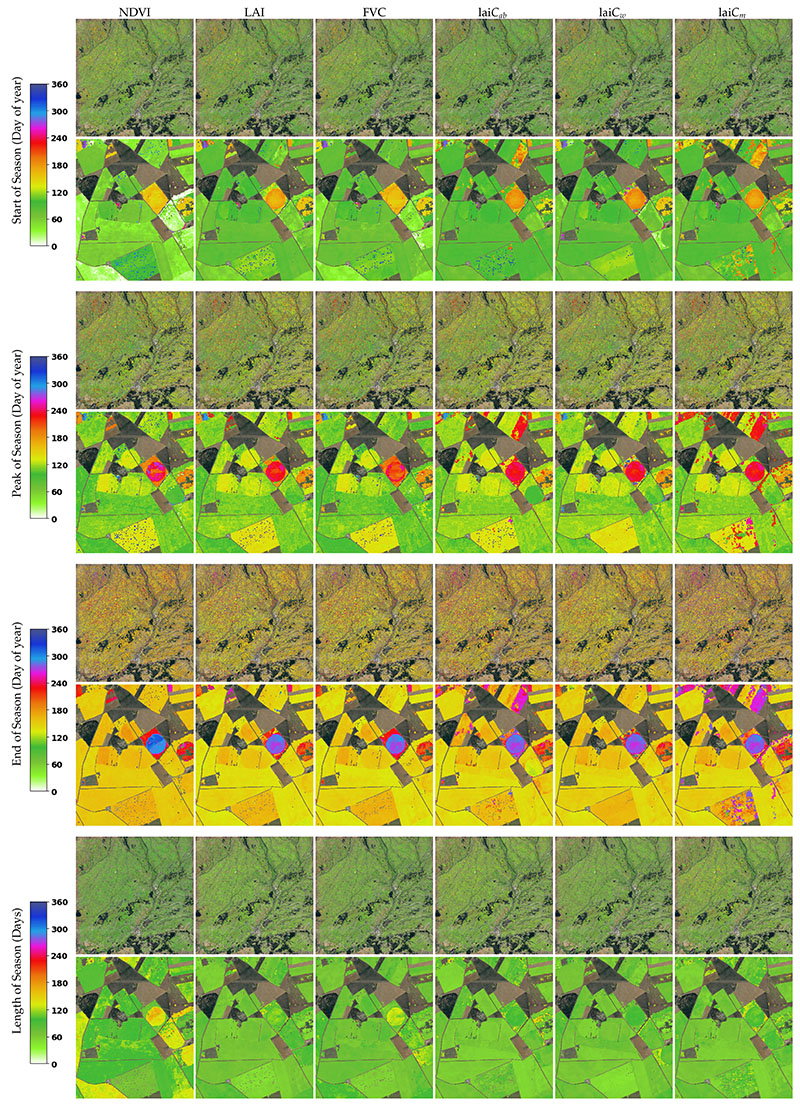
LSP values (SOS, POS, EOS, and LOS) of NDVI and crop traits for 30TUMin the year 2019 (Castile and Leon, Spain); underneath, a zoom-in of the study site. See Figure 2 for the geo-information.

The LSP metrics are calculated within a 1-year time window, with SOS typically occurring in spring and EOS in summer. It took merely 11–12 min per crop trait in GEE. Figure 7 shows the SOS, POS, EOS, and LOS from the tile 30TUM for the year 2019, generated from NDVI and the different crop traits. Although the LSP metrics can be calculated for any pixel, they are only meaningful over vegetated surfaces where phenology events occur. Therefore, we only demonstrate the maps of the croplands. As in Figures 6 and 7, the full tiles over the study site are shown as well as the zoom-ins underneath. The zoom-ins better enable inspecting similarities and differences. Overall, similar patterns appear for the different crop traits, suggesting that the same phenology was captured both by the crop traits and the NDVI. However, some local anomalies can also be observed, particularly for POS and EOS, with pixels returning much later dates. A closer inspection of their temporal profiles (Figure A1) revealed that for these pixels, two growth cycles emerged within one year. Since the LAI remained rather low, this could have been the effect of weed growth rather than the planting of another crop. Nonetheless, the LSP algorithm failed in identifying the first (main) growth cycle, but instead considered both growing cycles as one, resulting in strong shifts of the derived metrics.

### 3.4 Cropland-Based Phenology Trends

**Figure 8:**
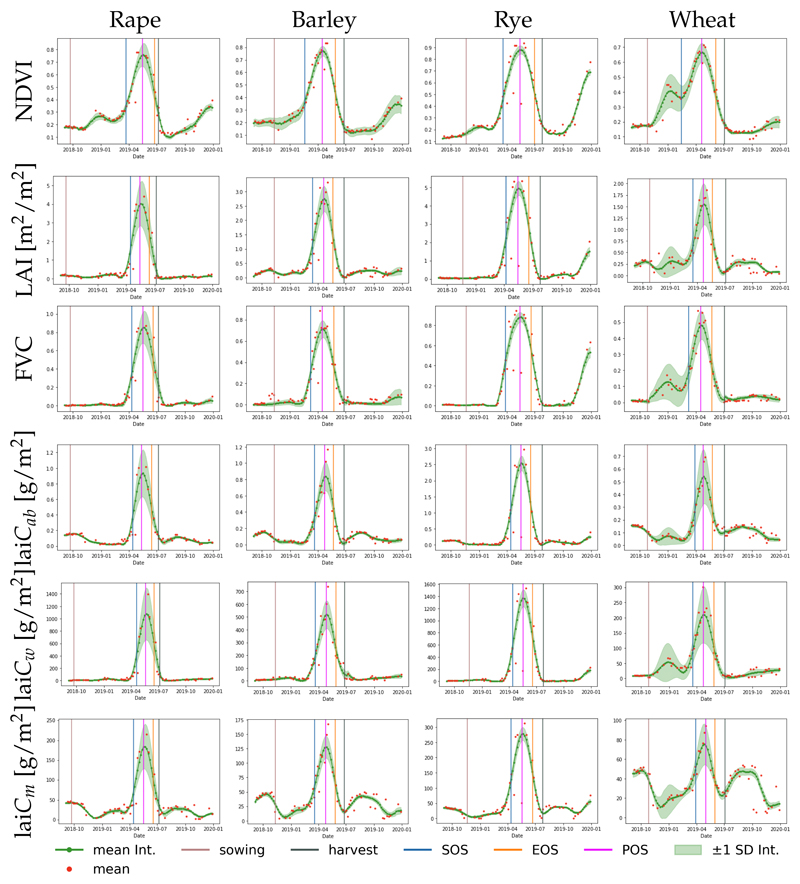
Time series profiles of the different crops: red dots indicate the original estimates, the green dots give the GPR-reconstructed retrievals. See Figure 2 for the geo-information. Derived SOS, EOS, and POS are indicated along with sowing and havest dates.

The maps in Figures 6 and 7 reveal that LSP metrics are influenced by the crop trait used for their calculations, suggesting the need for a closer inspection of the crop-specific development trajectories. Figure 8 shows the temporal profiles derived from NDVI and crop traits, as averaged for parcels of four crop types. The red dots indicate the original estimates, while the green dots give the GPR-reconstructed retrievals. Based on the red dots, it can be observed that retrievals lack temporal smoothness and can include outliers. Overall, all crop traits show a consistent seasonality pattern of one pronounced growth cycle with an increase, a peak, and then a decay. For all the profiles, the GPR gap-filling follows the S2 observations closely, confirming that GPR is an adequate fitting function. Moreover, apart from the parcel-averaged mean, the standard deviation (SD) is also provided. The SD suggests that temporal variability is more a function of crop type than of the trait. Especially for rape and wheat, a large variability can be observed. Conversely, the low SD of the rye field indicates the most homogeneous development of all crops over the season. The pronounced growth cycles match the double logistic curves closely, leading to the standard calculation of SOS, POS, and EOS, indicated by bars in Figure 8. It can be observed that the NDVI-derived curves are slightly broader than those of the traits, leading to a longer LOS as calculated by NDVI. Effectively, the calculated SOS and EOS fall in the middle of the green-up and decay phases, respectively, which is in accordance with the way the algorithm is designed. The SOS, for instance, is situated between leaf development and stem elongation, according to the corresponding phenological stages of the crops [77]. Sowing took place from about three (for NDVI) to a maximum of 6 months (for traits) before the calculated SOS, depending on crop type, Table 2 summarizes this. This large time gap between sowing and SOS can be explained by winter dormancy, when crop development remains in the vegetative phase. As soon as the crop resumes growth in early spring (main development phase of tillering), the plants start to green-up. Ideally, this point in time should be identified by SOS dates. EOS was correctly associated with late stages of the crop cycle such as ripening. Table 3 summarizes the EOS dates and the difference in days between the timing of the EOS and the harvest dates. On average, there was a time gap of 24 days between the harvest dates and the EOS for NDVI, a gap of 35 days for LAI, and of 30 days for laiCm, suggesting a relatively close correspondence. The difference of about one month comes from the principle of EOS calculation, using the right inflection point from the double logistic function. At this point in time, the crops will only have started senescence (i.e., an increase of non-photosynthetic biomass), but will not have reached full maturity yet. In addition, the harvest dates depend on several factors, such as local weather conditions or management decisions. Moreover, we can observe a temporal consistency among the crop types, with the closest correspondence of EOS and harvest dates for rape and the largest discrepancy for wheat.

**Figure 9:**
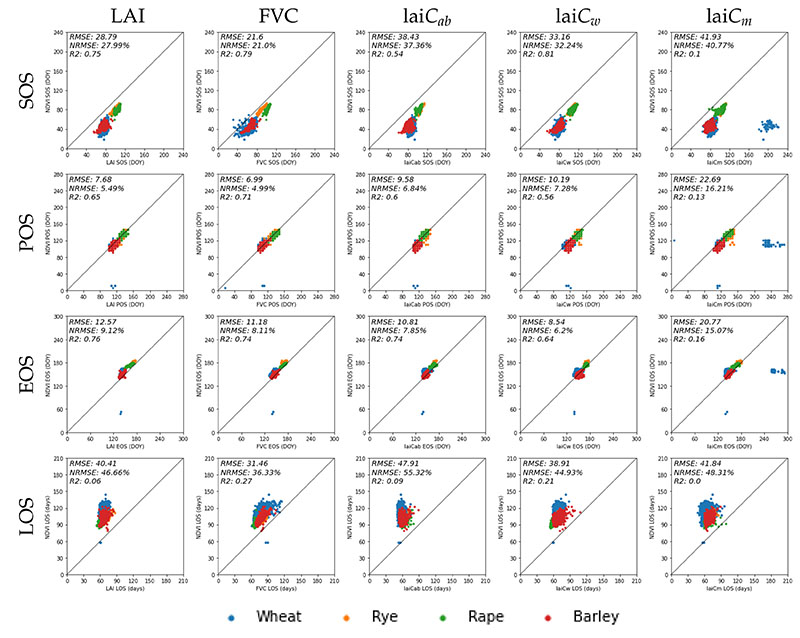
Comparison of Start of Season (SOS), Peak of Season (POS), End of Season (EOS) in day of the year (doy) as well as Length of Season (LOS), derived from NDVI and multiple crop traits. Case study site in the region of Castile and Leon, Spain in 2019. See Figure 2 for the geo-information.

In order to examine the robustness of the approach, the phenology metrics (i.e., SOS, POS, EOS, and LOS) derived from NDVI were plotted against those from the five crop traits for the cropland parcels of wheat, rye, rape, and barley (Figure 9).

Regarding SOS (Figure 9, first row), the traits give a systematically later DOY than NDVI for all crop types. On average, the difference is 33 days. The closest match is provided by the structural variable FVC showing the highest consistency with NDVI-derived metrics (NRMSE = 21%). The largest discrepancy can be found between NDVI and lai*C_m_* (NRMSE = 41%). In particular, this crop trait provides a strong mismatch for a cluster of wheat fields. The POS results suggest the closest correspondence of an LSP metric derived from NDVI and the traits (see Figure 9, second row). Though NDVI provides slightly earlier DOY for POS, relatively high correlations are presented for all traits (with max. R^2^ = 0.8 between FVC/lai*C_w_* and NDVI). Furthermore, a relatively high consistency between NDVI-derived and EOS DOY values calculated from the traits can be observed (Figure 9, third row). On average, the difference is only 12.77 days. The dates derived by FVC, LAI, and lai*C_ab_* are most similar to the EOS of NDVI. Due to a high discrepancy with some wheat fields, the doy of EOS derived from lai*C_m_* deviates most strongly from NDVI-based calculations (NRMSE = 15%). Regarding LOS (Figure 9, lower row), relatively strong deviations occur due to the mismatch provided by SOS calculations, with the highest discrepancy for lai*C_ab_* (NRMSE = 55%) and lai*C_m_* (NRMSE = 48%).

Overall, these trends suggest that lai*C_m_* may be least suitable for deriving phenology metrics. In contrast, lai*C_ab_* seems to provide the most realistic and smooth seasonal courses, with rather low variability. The closest match with the NDVI-based metrics is given by LAI and FVC (see discussion section).

## 4 Discussion

With the ambition of automating the mapping of trait-based LSP metrics over croplands, an optimized machine learning strategy was developed for processing on GEE. The end-to-end framework consists of the following key steps: (1) optimizing the hybrid GPR retrieval models of crop traits to facilitate their implementation on GEE; (2) time series processing of the traits and gap-filling through GPR fitting, and finally, (3) calculation of common phenological metrics. The three main steps are further discussed below, followed by encountered limitations, challenges, and future opportunities of the workflow. As the major part of the workflow is streamlined in GEE, we present a paradigm shift that moves away from traditional image analysis using desktop software up to cloud-based processing.

### 4.1 Hybrid Retrieval of Crop Traits from L2A S2 Data

Driven by the need to develop light GPR retrieval models to enable the quantification of crop traits on GEE, a key achievement was the substantial improvement through the AL strategy. Using the EBD method for the optimal reduction of the training datasets led to excellent performances with relatively few simulations for training GPR models, confirming earlier experiences with AL [31,51,52,60]. The increase in retrieval accuracy can be explained by the positive effects of this intelligent sampling method, which decreases redundancy but keeps the variability of reflectance datasets. It allows us to cover the defined range of the variable, while avoiding the overfitting problem at the same time. Although the training datasets were heavily reduced as opposed to the full dataset (75% or more), mapping performances were superior for all variables when using EBD-optimized models. These results suggest that GPR models benefit from training data quality rather than quantity, as also found by Berger et al. [51]. Note that the AL sampling selection is run against in situ field data. Thus, it is essential that this reference dataset covers a sufficiently broad range of crop growth stages. Nonetheless, the collection of high quality field data remains a critical part of the retrieval algorithm development and should be pursued in future studies. Furthermore, the retrieval models need to be adapted to diverse spectral surfaces in order to secure the generic processing of all pixels. This was realized by extracting non-vegetated spectra (i.e., bare soil, water, man-made surfaces, etc.) from the S2 scenes and adding them to the training dataset. Moreover, our results suggest that the GPR models retrieve traits at the canopy level more accurately than at the leaf level. This can be explained by the usage of LAI for upscaling, thus accounting for the influence of the canopy structure on the recorded spectral signal [78].

### 4.2 Spatiotemporal Crop Trait Processing on GEE

Once the GPR crop trait models were implemented on GEE, in principle, these products can easily be retrieved anywhere in the world and at any time. The advantages of GEE as an image processing platform are unprecedented; GEE has opened a new big data paradigm for the storage and analysis of open-access EO data at a scale that is unfeasible with desktop processing machines [29]. To provide light retrieval models for GEE, the following adaptations were introduced in our study: (1) We expanded the formulation of standard GPR, (2) aggregated all terms independent of the pixels’ spectral information that can be pre-calculated to avoid the repeating cumbersome operation for each pixel, (3) performed data manipulation that can be carried out using an *image* data type format before moving to *array* data type, (4) implemented GPR regression into a matrix algebra formulation, and (5) converted the results back to *image* format, adding coordinate information that is mandatory for mapping [31]. With this framework in place and a pre-trained GPR model at our disposal, it can be integrated into the developed workflow after substituting the new hyperparameter values and the corresponding training samples as well as normalization matrices. Although the latter still requires manual implementation, it is foreseen to automate and optimize all steps, which will eventually allow for an intuitive import of GPR models on GEE.

A subsequent key step of the processing is the resolved spatiotemporal aspect. The proposed gap-filling strategy yielded promising and consistent results. The examples of cloud-free crop trait collections demonstrate the great potentials of the GPR regression technique as a gap-filler. In case the persistence of cloud cover exceeds two months, the gap-filling method becomes less reliable as rapid vegetation dynamics might be lost or smoothed. Alternative approaches based on multisensor time series fusion should be taken into consideration. An example is the multi-output Gaussian process regression proposed in Pipia et al. [37], though its implementation in GEE is still missing and represents a challenge for future development. Two remarks are worth mentioning. First, the gaps were not only filled here, but in fact all the tiles were reconstructed according to the GPR fitting, avoiding outliers in the temporal profiles. It also implies that reconstruction can be achieved for any time interval, e.g., the production of the crop traits on a daily basis. A second remark is that GPR provides associated uncertainty intervals (not shown here), which enables us to track the confidence of the reconstruction.

On the downside, being a kernel-based machine learning method, GPR processing comes with computational costs in its conventional usage. While processing time for a single pixel time series is negligible (i.e., on the order of 0.1 s), computational demand for large images significantly increases. It makes this method impractical when aiming to process data streams of complete S2 tiles, which contain over 30 M pixels at 20 m resolution. Therefore, computationally efficient alternatives must be sought in order to deal with such big data. We followed the workaround proposed by Belda et al. [64], whereby the GPR *θ* hyperparameters were pre-calculated per crop trait. With an S2 demonstration case, Belda et al. [64] showed that LAI time series performances stayed alike in terms of RMSE when compared against the default per-pixel optimized setting, i.e., without pre-calculation. The approach first requires optimizing the GPR hyperparameters *θ* over a limited subset of crop pixels, either homogeneous or heterogeneous, and then fixing their value for the GPR fitting function. Although this leads to a slight loss in accuracy, it gains tremendously in run-time and therefore allows us to process entire time series of S2 tiles onto cloud-computing platforms. As such, it allowed us to process the entire catalogue of the crop traits for a single tile within reasonable time, on the order of 2 h for each crop trait per year. As a result, this led to reconstructed spatiotemporal continuous maps in GEE. At the same time, as the GPR model follows the general temporal trend, there is no need to apply a smoothing function. Hence, consistent estimates are generated without outliers. While such consistency favors the calculation of LSP metrics, it also bears the consequence that sudden events such as anomalous crop development through abiotic [79] or biotic stresses [80], or short phenological events such as flowering, are smoothed out. It thus suggests that the GPR-generated data stream may become less suitable for the detection of sudden changes.

### 4.3 LSP Metrics Estimation

Thanks to the production of spatiotemporal continuous data streams, LSP metrics can be processed in the final step. Following the approach of Li et al. [67], calculation was straightforward and fast, leading to the mapping of SOS, EOS, POS, and LOS. Typically, these LSP metrics are derived from indices such as the NDVI (see [11,81] for reviews).

However, the usage of two-band indices faces limitations such as a reduced exploitation of the spectral information content. In addition, indices may be influenced by geometry effects (i.e., the sun zenith angle) as demonstrated by Ma et al. [82] for NDVI temporal profiles, or by soil brightness [83]. More fundamentally, the NDVI is only an indicator of greenness, lacking a quantitative meaning. To overcome these index-related drawbacks, we introduced a few novelties in our study. First, we retrieved a suite of quantifiable crop traits, which are supposedly more robust than vegetation indices. These traits provided a tighter shape for the seasonal courses, which may be due to the weaker influence of external factors compared to NDVI. The GPR retrieval models exploit ten S2 bands based on generic training datasets as produced by a coupled leaf-canopy RTM that is configured by multiple biochemical leaf, structural canopy, and also geometry variables. Hence, the RTM takes the variability of illumination conditions into account. A second novelty is that we processed an S2 tile into traits with a machine learning fitting algorithm (GPR) on GEE. Earlier studies have demonstrated that GPR outperformed other fitting functions in the reconstruction of time series data streams [37,38], providing realistic gap-filled, spatiotemporal continuous data.

Regarding the usage of traits as opposed to NDVI, the following general findings can be derived: (1) lai*C_m_* often detected a second smaller peak before or after the crop cycle. While that may indicate non-photosynthetic (dry) vegetated material, this trait appeared to be unstable for reliable LSP metric calculation, usually providing a single pronounced peak (POS). Hence, this suggests that this trait is less suitable for LSP calculation. (2) Metrics derived from FVC and LAI most resembled those from the NDVI approach and identified a pronounced growing cycle. Since PROSAIL-based FVC and LAI variables are sensitive to vegetation greenness, they may behave similarly to NDVI. (3) Although these traits show similar patterns, laiCab was the most realistic for LSP metric derivation. It followed a smooth course over the season without deriving a second or a third peak (such as lai*C_m_* or NDVI), and provided the lowest variability. Moreover, the combination of a leaf-level biochemical variable (*C_ab_*) with a biophysical structural variable (LAI) may be the optimal synergy to realistically derive the actual crop development.

An interesting finding is that some traits identified a second yet smaller cycle. It remains to be verified whether this is due to crop rotation or rather due to the occurrence of weeds. Nevertheless, the used approach to calculate the LSP metrics, i.e., the double logistic function, was unable to distinguish between the two cycles. Follow-up research is foreseen to explore whether the function can be operated more flexibly, e.g., to force falling SOS and EOS more to the tails of the curves. Some adaptations were proposed to tune the SOS and EOS more closely towards the crop calendar [84,85], e.g., estimating the phenological transition dates by using local extremes for the rate of change in the curvature of the fitted double logistic model (Zhang et al. [14]). In time series software packages that calculate LSP metrics, thresholds can be set. For instance, both in TIMESAT [86] and DATimeS, the detection of SOS and EOS can be tuned based on conventional threshold methods, analogous to [86–91]. Therefore, threshold values can be set by searching the seasonal trajectories for each defined season and tuning the definition of the amplitude. This allows for a calibration stage, e.g., by making use of PhenoCam images, carbon flux measurements, or manual phenology observations [92,93].

Furthermore, it may also be worth exploring more irregular temporal curves, e.g., with less pronounced or multiple peaks. This may especially be necessary when multiple crops are cultivated within one year. For these cases, alternative fitting functions should be explored as well in GEE, including for instance the hyperbolic tangent function. This function is similar to the double logistic, but with the addition of a seventh parameter [77,94]. Though calibration will enable a closer match of the EOS with the harvest date, it also introduces additional challenges for generic processing on GEE. This research avenue will be further explored in follow-up studies.

### 4.4 Limitations, Challenges, and Future Opportunities

While this study forged ahead towards generic LSP mapping onto a cloud-computing environment, the pursued approach poses some limitations and challenges. They are briefly listed below, followed by proposed solutions.

(1) The GPR models need to be kept sufficiently small to avoid memory problems in GEE. At the same time, the developed trait retrieval models may be less robust than expected. Validation was only performed at one site and one point in time, i.e., during summer. Hence, the robustness of the models throughout the full season remains to be evaluated.

(2) The occurrence of cloudy periods (particularly during the winter months) may lead to larger data gaps, limiting highly accurate retrievals throughout the entire year. To circumvent this, GPR fitting provided spatiotemporally consistent and smoothed data streams. The sole drawback of this approach is that pre-computed hyperparameters are required to enable fast processing. This not only implies that those hyperparameters first need to be generated based on representative data, but it is also to be questioned whether the introduced end-to-end approach is generically valid. Further research is required to evaluate its robustness. For instance, other gap-filling methods may be as flexible as GPR without relying on hyperparameter tunings. Promising experiences were obtained with the Whittaker fitting method [38]. In addition, note that part of the processing was performed with ARTMO and DATimeS software tools (desktop processing requiring a Matlab license), and only the final models were subsequently incorporated in GEE. Further research could explore the use of open-source alternatives to integrate the full processing chain in GEE.

(3) When aiming to use EOS as an indicator for cropland harvest detection, the applied double logistic method may need some adaptations: while the algorithm was optimized for running on GEE [67], EOS should be tuned to rather fall at the tail of the senescence stage. Additional efforts are required to enhance the sensitivity of the algorithm towards identifying multiple cycles within a year. Despite all limitations, we demonstrated that crop traits can be efficiently quantified over multiple years and anywhere in the world using the GEE computing platform. With this, GEE opens doors for higher-level processing, such as gap-filling based on the global time series of Sentinel-2 satellite data, offering deca-metric spatial resolutions and high temporal repetition times. Land surface phenology metrics estimated from these dense time series can be incorporated into precision farming management activities [95], enabling crop monitoring and thus supporting agricultural decision systems to mitigate the risk of food shortage. By providing such information, our proposed workflow could assist in the progress towards the Agriculture 5.0 era [96], supporting the evolution of precision farming with cutting-edge technologies. For instance, timely information about crop development is of high interest for arid and semi-arid environments, as can be found in Spain in some areas where high rainfall variability occurs, leading to inter-annual fluctuations in primary production [79]. A comparison of actual cropland profiles with those of other seasons or with the long-term average may indicate trends or anomalies, which could trigger timely measures and on-field interventions to secure agricultural productivity.

## 5 Conclusions

This study presents an integral workflow for the retrieval of crop traits and their associated land surface phenology metrics in the GEE cloud platform. We propose the following S2 BOA end-to-end processing chain: (1) the building of hybrid GPR retrieval models of crop traits optimized with AL, (2) migration of these models onto GEE, (3) generation of spatiotemporally continuous maps and time series with the use of gap-filling through GPR fitting, and (4) calculation of LSP metrics. Predicted mean and cloud-free maps were generated for an agricultural test site in the region of Castile and León, Spain. Subsequently, LSP metrics were obtained over this area. Comparison to crop calendar data and NDVI reference products proved the successful implementation of the pursued workflow. While the metrics derived from FVC and LAI captured a pronounced growing cycle, behaving similarly to NDVI, the most sensitive LSP metric derivation was lai*C_ab_*; the combination of a leaf-level biochemical variable (*C_ab_*) with a biophysical structural variable (LAI) may be the optimal strategy for realistically deriving the actual crop development. However, this has to be further investigated using data of exact phenological stages. As a future perspective, this work opens the possibility to incorporate uncertainty estimates on GEE, being one of the most interesting characteristics of GPR. Follow-up research is required to evaluate the robustness of hyperparameter pre-computation and overall global validity using multiple reference datasets. Overall, our work provides a roadmap towards the automatic derivation of quantitative and spatiotemporally continuous crop phenology information at a global scale. Over the next decades, we expect an abundance of space-based open-access satellite observations, which will further increase GEE capabilities and thus support the monitoring and management practices of cultivated areas.

## Supplementary Material

The following link contains a repository with demo codes of the different procedures used in this paper https://github.com/msalinero/GEEGPRPhenoDemos.git.

Supplemental tables

## Figures and Tables

**Table 1:** Goodness-of-fit results of estimated vs. measured crop traits at the MNI site. Results are given for the GPR model trained with the original datasets (Full) compared to the EBD-optimized datasets (EBD). Units of RMSE for *C_ab_* in (μg/cm^2^), for *C_w_* in (cm), for *C_m_* in (g/cm^2^), for LAI in (m^2^/m^2^), for lai*C_ab_*, lai*C_w_*, and lai*C_m_* in (g/m^2^). NRMSE in %.

Variable	C_ab_		C_w_		C_m_		LAI		laiC_ab_		laiC_w_		laiC_m_	
Dataset type	Full	EBD	Full	EBD	Full	EBD	Full	EBD	Full	EBD	Full	EBD	Full	EBD
RMSE	4.2775	4.1869	0.0067	0.0021	0.0013	0.0009	0.4569	0.3695	0.3034	0.1907	142.0915	103.68	53.5665	42.1821
**NRMSE**	19.1472	18.7417	53.9326	16.8862	26.1028	18.2138	12.3714	10.0056	14.1497	8.8947	16.6034	12.1158	18.7811	14.7895
**R^2^**	0.8079	0.8143	0.2219	0.5970	0.1631	0.6590	0.8896	0.9139	0.8629	0.9253	0.8219	0.8490	0.5910	0.7372

**Table 2:** Mean values of Start of Season (SOS) in day of year and its mean difference from the sowing date (Diff) in days, for each crop trait and crop type. The smallest differences are written in bold.

	SOS_NDVI_	Diff_NDVI_	SOS_LAI_	Diff_LAI_	SOS_FVC_	Diff_FVC_	SOS_laiCab_	Diff_laiCab_	SOS_laiCw_	Diff_laiCw_	SOS_laiCm_	Diff_laiCm_
**Wheat**	44	**110**	75	141	67	133	88	154	78	144	87	153
**Rye**	78	**118**	96	136	87	127	104	144	103	143	102	142
**Rape**	82	**181**	105	204	101	200	103	202	109	208	104	203
**Barley**	48	**98**	73	123	67	117	81	131	81	131	78	128

**Table 3:** Mean values for End of Season (EOS) in day of year and its mean difference from the harvest date (Diff) in days, for each crop trait and crop type. The smallest differences are written in bold.

	EOS_NDVI_	Diff_NDVI_	EOS_LAI_	Diff_LAI_	EOS_FVC_	Diff_FVC_	EOS_laiCab_	Diff_laiCab_	EOS_laiCw_	Diff_laiCw_	EOS_laiCm_	Diff_laiCm_
**Wheat**	156	**30**	142	44	143	43	145	41	148	38	151	35
**Rye**	180	**27**	170	37	171	36	168	39	170	37	172	35
**Rape**	175	**13**	165	23	170	18	165	23	168	20	168	20
**Barley**	148	29	140	37	143	34	142	35	149	**28**	146	31

## Data Availability

Not applicable.
