# Supplementary material for: Monitoring Cropland Phenology on Google Earth Engine Using Gaussian Process Regression
Source: Remote Sens (Basel). Author manuscript; Available in PMC 2022 Sep 7. (PMC7613380; doi:10.3390/rs14010146)
Supplement: Supplemental tables [file EMS152679-supplement-Supplemental_tables.pdf]

Appendix A.

Table A1. Parameterization of leaf (PROSPECT-4) and canopy (4SAIL) parameters, with the notations, units, ranges, and distributions of inputs used to establish BOA synthetic reflectance databases. $\bar{x}$: mean, SD: standard deviation. LHS: Latin hypercube sampling.

Model Variables		Units	Range	Distribution
Leaf variables: PROSPECT-4				
N	Leaf structure parameter	unitless	1.3–2.5	Uniform
C_{ab}	Leaf chlorophyll content	(µg/cm^2)	5–75	Gaussian ($\bar{x}$: 35, SD: 30)
C_m	Leaf dry matter content	(g/cm^2)	0.001–0.03	Gaussian ($\bar{x}$: 0.005, SD: 0.001)
C_w	Leaf water content	(cm)	0.002–0.05	Gaussian ($\bar{x}$: 0.02, SD: 0.01)
Canopy variables: 4SAIL				
LAI	Leaf area index	(m^2/m^2)	0.1–7	Gaussian ($\bar{x}$: 3, SD: 2)
α_{soil}	Soil scaling factor (brightness)	unitless	0–1	Uniform
ALA	Average leaf angle	(°)	40–70	Uniform
HotS	Hot spot parameter	(m/m)	0.01	-
skyl	Diffuse incoming solar radiation	(fraction)	0.05	-
FVC	Fractional vegetation cover	(fraction)	0.05–1	-
Illumination/ observation conditions: 4SAIL and 6SV				
θ_s	Sun zenith angle	(°)	20–30	Uniform
θ_v	View zenith angle	(°)	0	-
ϕ	Sun-sensor azimuth angle	(°)	0	-

Table A2. Averaged hyperparameters estimated using the global approach: l defines the gap-filled time series smoothness, σ_f is the amplitude scaling factor, and σ_n accounts for the noise variance.

	NDVI	LAI	FVC	laiC$_{ab}$	laiC$_w$	laiC$_m$
l	32.917	28.2361	31.6638	28.1263	28.0052	29.0619
σ_f	0.1818	0.8967	0.2189	0.2333	176.4995	38.9518
σ_n	0.0552	0.3156	0.0703	0.0831	63.9533	13.1938

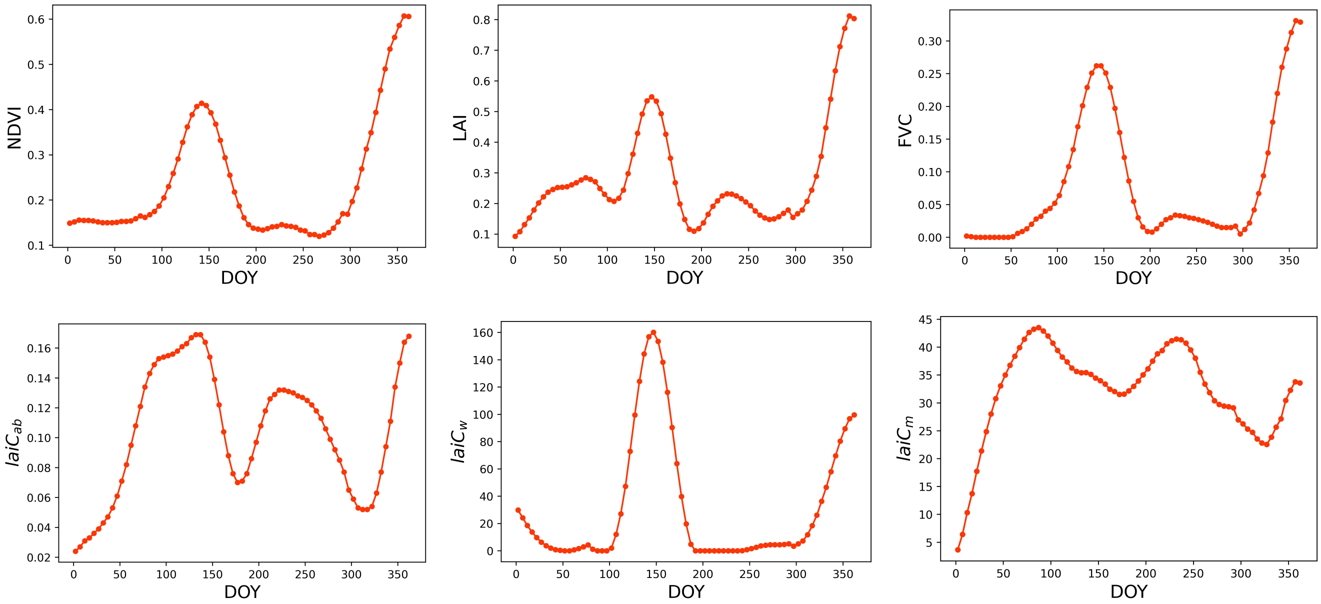

Figure A1. Time series profiles of crop traits for a wheat pixel presenting double seasonality.
